# Comparison of the Permeability of Potential Ballast Rocks from Northern Rio de Janeiro State under Different Fouling Rates after Sodium Sulfate Attack

**DOI:** 10.3390/ma16103806

**Published:** 2023-05-18

**Authors:** Rodolpho N. Souza, Gustavo de Castro Xavier, Kelly de Oliveira Borges da Costa, Jonas Alexandre, Rogério P. Ribeiro, Afonso R. G. de Azevedo

**Affiliations:** 1 LECIV—Civil Engineering Laboratory, UENF—State University of Northern Rio de Janeiro, Av. Alberto Lamego, 2000, Campos dos Goytacazes 28013-602, Brazil; rodolpho.n@hotmail.com (R.N.S.);; 2 Geotechnical Engineering Department, São Carlos School Engineering, USP—State University of São Paulo, Av. Trabalhador São-Carlense, 400, São Carlos 13566-590, Brazil

**Keywords:** meta granites and gneiss, rail ballast fouling, hydraulic conductivity, Micro-Deval, porosimety, sodium sulfate

## Abstract

This paper aimed to analyze the reduction in the ballast layer permeability simulated in a laboratory in saturated conditions by the presence of rock dust as a contaminant of three types of rocks explored in different deposits in the northern region of the state of Rio de Janeiro, Brazil, through laboratory testing relating the physical properties of rock particles before and after sodium sulfate attack. Sodium sulfate attack is justified by the proximity of some sections of the planned EF-118 Vitória-Rio railway line to the coast and of the sulfated water table to the ballast bed, which could degrade the material used and compromise the railway track. Granulometry and permeability tests were performed to compare ballast samples with fouling rates of 0, 10, 20, and 40% rock dust by volume. A constant head permeameter was used to analyze hydraulic conductivity and establish correlations between the petrography and mercury intrusion porosimetry of the rocks, namely two types of metagranite (Mg1 and Mg3) and a gneisse (Gn2). Rocks, such as Mg1 and Mg3, with a larger composition of minerals susceptible to weathering according to petrography analyses, tend to be more sensitive to weathering tests. This, in conjunction with the climate in the region studied, with average annual temperature and rainfall of 27 °C and 1200 mm, could compromise track safety and user comfort. Additionally, the Mg1 and Mg3 samples showed greater percentage variation in wear after the Micro-Deval test, which could damage the ballast due to the considerable changeability of the material. The mass loss caused by abrasion due to the passage of rail vehicles was assessed by the Micro-Deval test, with Mg3 (intact rock) declining from 8.50 ± 1.5 to 11.04 ± 0.5% after chemical attack. However, Gn2, which exhibited the greatest mass loss among the samples, showed no significant variation in average wear, and its mineralogical characteristics remained almost unchanged after 60 sodium sulfate cycles. These aspects, combined with its satisfactory hydraulic conductivity rate, indicate that Gn2 is suitable for use as railway ballast in the EF-118 railway line.

## 1. Introduction

Given the increased demand for the efficient transportation of high-density material and the growth of agribusiness nationwide, the predicted expansion of the Brazilian rail network is encouraging and has prompted considerable investment in the sector, considered one of the most efficient and logistically promising modes of transport. An example of this is the 577 km-long EF-118 railway line that will link the port in Vitória, Espirito Santo state, to the port of Rio de Janeiro [1].

However, there is growing concern regarding the origin and quality of components for this type of venture, since track ballast is a key formative element of railway lines [2] and the grading and mechanical strength of the rock determine ballast behavior [3]. As such, it is important that the material used be submitted to petrographic analysis to assess the properties of its particles [4].

Given that railway ballast is subject to mechanical action from the type of traffic it is exposed to, maintenance costs, track stability and hydraulic conductivity may be directly affected by the gradual and continuous degradation of the track bed, which can compromise the mechanical properties of the ballast material, requiring more frequent track maintenance [5]. 

The surface of the ballast bed should be as flat and uniform as possible, with thickness varying from 30 to 40 cm. Particle size distribution significantly influences track deformation behavior, since density and friction angle decline as particle uniformity increases [6]. 

Track ballast with a more varied granulometry has fewer voids, making the bed more resistant. However, its drainage efficiency may be compromised by fouling (contamination) through the accumulation of fine particles in these voids [7].

An intermediate grading is ideal for railway ballast, which should also provide satisfactory drainage, density, and shear strength for the track bed. In Brazil, NBR standard [8] stipulates a particle size of 12.5–50 mm for ballast rock.

According to [9], the response of ballast to permeability and degree of saturation varies. Percolated water may favor particle rupture and influence ballast settlement, which could directly affect vehicle traffic. 

Track operation causes the fracture and abrasion of ballast particles, resulting in excess production of rock dust [10]. This reduces ballast permeability and subgrade infiltration, which can compromise the safety and riding comfort of moving trains [11].

Major causes of track stiffness include ballast grading and the breaking of sharp corners of the aggregate that comprises the track bed. As observed by [12], this effect can compromise ballast permeability and cause structural problems.

According to [13], ballast breakdown is the most important type of contamination (fouling), which compromises its permeability. Track maintenance work indicates that the particle size of granite ballast can decrease by up to 50% of its unit mass and up to 10% for limestone ballast [14]. 

Thus, the permeability of railway ballast declines rapidly as fouling increases [15].

The main objective of this article was to assess the water table in the region where the railway line will be laid, since it could permeate the bottom of the ballast layer due to the large volume of rainfall recorded between November and March.

In order to obtain a high-performing aggregate with efficient hydraulic percolation for use as railway ballast, it is increasingly important to investigate particle behavior and drainage capacity. 

As such, the present study analyzed reduced permeability in wet railway ballast with fouling rates of 10%, 20%, and 40%, caused by rock dust from loading and local weathering, using laboratory tests and establishing correlations between the petrographic properties of the material and constant head testing.

Properties, such as petrography, porosimetry, and hydraulic conductivity, were analyzed in rocks with the potential for application as ballast in the EF-118 railway line before and after sodium sulfate attack, in order to demonstrate the main changes and effects on the rock particles due to weathering. 

The EF-118 will connect two states and 25 municipalities, covering 577.8 km, with 100 km of this in the municipality of Campos.

Its proximity to the coast and the fact that the water table in the area is close to the surface mean the line may be exposed to sulfated water [16,17]. The rocks studied are extracted from an area near the planned railway line and contain minerals susceptible to sodium sulfate attack, which may alter the properties of these rocks.

## 2. Materials and Methods

The Mineral Resources Department of Rio de Janeiro State (DRM-RJ) compiled a geological map, characterizing its regions into different sections. The rocks in the present study were collected from the North-Northeast section to the north of the Paraíba do Sul River. This area is predominated by gneisses and charnockites, structurally classified as massive and sometimes banded, in addition to garnet crystals.

A case study was conducted, using three samples of metamorphic rock extracted from deposits in Rio de Janeiro state (RJ), Brazil. 

Metamorphic rocks arise from the transformation of existing rock, which undergoes chemical and structural changes due to physical and chemical conditions below the Earth’s surface, such as differences in pressure, temperature, and fluids, usually water. The most widely used metamorphic rocks are marble, quartz, gneiss, slate and phyllite [18].

The aggregates used in our study were metagranites (Mg1 and Mg3) and gneisse (Gn2), with approximately 10 kg of each collected directly from the tailing piles at quarries. 

This study is important because of the area in which EF-118 will be located. According to the Environmental Impact Assessment (EIA) for the São João da Barra Industrial District (2011), climate in the region is hot and semihumid (four to five dry months) due to factors, such as location, topography and air mass [16]. This tropical climate produced an average annual temperature of 23.9 °C in 2021, with the highest (27 °C) and lowest (21.2 °C) average monthly temperatures recorded in February and July, respectively, according to the National Institute of Meteorology [19].

In addition to the fluctuating high temperatures in the region, its rainfall is also a relevant factor for ballast used in EF 118, with levels varying from 24 to 202 mm in the driest (June) and wettest months (December), respectively. Total annual rainfall in the region is around 1200 mm, with approximately 130 days of rain per year [16].

The highest relative humidity (RH) was measured in December (79.07%) and the lowest in September (70.77%). On average, December had the rainiest days (17.73) and August the fewest (5.67), with the potential to increase fouling in the line. According to [19], average wind speed in the region in 2021 varied by 6.36 m/s for maximum gusts, with an hourly speed of 3.49 m/s, thereby compromising its permeability.

Thus, climate in the coastal region can accelerate ballast degradation through weathering due to the proximity of the sulfated water table to the surface and the high rainfall.

Pellegrino (2007) [18] characterized weathering susceptibility in crystalline-metamorphic rocks and found that the effects were amplified along the thermal-metamorphic contact belt. There was considerable geomechanical decomposition of the crystalline-metamorphic rocks, confirming the effect on the geomechanical behavior of the rock mass. 

Thus, our study is relevant due to the use of rocky material as railway ballast with a significant presence of weathering agents in the planned location of the EF-118 railway line. 

The particle size distribution of the ballast was assessed in accordance with [20] and [8], which stipulate ballast standards, as shown in Table 1.

Particle size and fouling rate for large aggregates are established considering the material retained in the largest number of sieves with a mesh size between 38 and 12.5 mm. Additionally, the rock dust used to evaluate ballast permeability was sieved through 6.7 to 0.075 mm mesh sieves, using approximately 6 kg of each rock type.

The level of fouling was determined based on the fouling index (F1), whereby values lower than 1 indicate clean ballast, 1–10 reasonably clean, 10–20 moderately clean, 20–40 fouled ballast, and greater than 40 highly fouled, considering the large mesh sieves, as shown in Equation (2).

According to this index, the need to clean ballast is established by adding the indices obtained for the percentage of material that passed through the sieves (*FI*), with a result higher than 80% indicating the need for immediate cleaning.

(1) $$FI=\left(0.4\;F19\right)+\left(0.3F6.7\right)+\left(0.2F1.18\right)+\left(0.1F0.15\right)$$

(2) $$FI19=\frac{\left(\%\;of\;material\;passing\;the\;19\;\mathrm{mm}\;sieve\right)\times 100}{27}$$

(3) $$FI6.7=\frac{\left(\%\;of\;material\;passing\;the\;6.7\;\mathrm{mm}\;sieve\right)\times 100}{18}$$

(4) $$FI1.18=\frac{\left(\%\;of\;material\;passing\;the\;1.18\;\mathrm{mm}\;sieve\right)\times 100}{11.5}$$

(5) $$FI0.15=\frac{\left(\%\;of\;material\;passing\;the\;0.15\;\mathrm{mm}\;sieve\right)\times 100}{27}$$

where FI is the sum of the percentages of material that passed through the different sieve sizes and FI19, FI6.7, FI1.18, and FI0.15 are the percentages of material that passed through the 19, 6.7, 1.18, and 0.15 mm sieves, respectively.

Petrography was performed to assess the mineral changes, texture, nature, and classification of the rocks. The test was conducted in line with [21], using 10–20 cm rock fragments of each of the three rock types studied.

The material was analyzed using a Carl Zeis Axioskop 40 polarizing microscope belonging to the Mineral Technology Laboratory at the Espirito Santo Mineral Technology Center (CETEM/ES) in Cachoeiro de Itapemirim, Espirito Santo State (ES), Brazil.

The rock material was analyzed in the laboratory before and after accelerated degradation by sodium sulfate, using 60 submersion and drying cycles, in line with [8].

The number of cycles was established based on the minimum of 5 recommended by [8] and the objective of assessing degradation across the largest number of cycles. Sodium sulfate attack consisted of 61 days of wetting, cooling, and drying cycles. 

The physical indices of each of the samples were determined based on apparent specific gravity (Equation (6)), apparent porosity (Equation (7)), and water absorption (Equation (8)). 

The text was conducted according to Annex B of the guideline [8], using 10 rocks of each of the types analyzed (Mg1, Gn2, and Mg3) with equivalent diameters between 5 and 7 cm.

The physical indices were determined before and after sodium sulfate attack.

(6) $$\rho a=\frac{Wdry}{\left(Wwet-Wsub\right)}$$

(7) $$\eta a=\frac{\left(Wwet-Wdry\right)}{\left(Wwet-Wsub\right)}\;\times \;\;100$$

(8) $$\alpha a=\frac{\left(Wwet-Wdry\right)}{\left(Wdry\right)}\;\times 100$$

where (𝜌𝑎) is the apparent specific gravity (g) of the aggregate; (*Wdry*) the weight of the dry sample (g); (*Wwet*) the weight of the wet sample (g); (𝑀𝑠𝑢𝑏) the weight of the submerged sample (g); (𝜂𝑎) apparent porosity (%), and (⍺𝑎) water absorption (%).

Data on rock porosity were obtained using an Autopore IV 9500 mercury porosimeter (Micromeritics Instrument Corporation, Norcross, GA, USA). Mercury intrusion into rocks smaller than 9 mm was analyzed at controlled pressures between 0 and 33,000 psi (228 MPa), covering a pore diameter range of 0.5–360 μm, the pressure needed for mercury to intrude into rock, which is inversely proportional to pore size. This test was conducted to determine whether pores in larger particles (12.5–50 mm) influence permeability after sodium sulfate attack. 

The abrasion test measures the relative quality and durability (resistance to weathering) of mineral aggregates subjected to abrasion and impact. The results obtained in the above tests provide a measure of toughness and abrasion resistance, as well as specific information on the durability of a sample aggregate ground with steel balls in the presence of water [22,23]. 

Samples are prepared by combining different weights of particle size fractions, processed in the devices according to the relevant test methods and then washed in a specific sieve to determine percentage loss to abrasion.

The Micro-Deval test was used to evaluate the wear resistance and durability of the material under unfavorable exposure conditions, using a [24] 1500 ± 5 g sample with particle size between 9.5 and 16 mm, submitted to 100 rpm com 5 kg steel balls and 2 L of water, in accordance with [24]. The test measures the polishing resistance of the aggregate, quantified by the percentage weight loss of the material analyzed [23].

The test was conducted at the Civil Engineering Laboratory of the State University of Northern Rio de Janeiro (UENF), using representative samples of the aggregates studied here to assess accelerated abrasion, before and after sodium sulfate attack, when exposed to a wet environment, such as the region of the planned EF 118 railway line. The test was carried out to establish the minimum fouling percentage for the permeability test, with approximately 10% weight loss, and to relate it to the mineralogical composition. 

A constant head permeameter is used for laboratory simulations whereby particles are submerged under constant water flow, which percolates the voids in the particulate. This simulates flooding conditions due to the heavy rainfall which the ballast particles may be exposed to. 

These devices are widely used to determine saturated hydraulic conductivity (K) under laboratory conditions [25].

**Figure 1:**
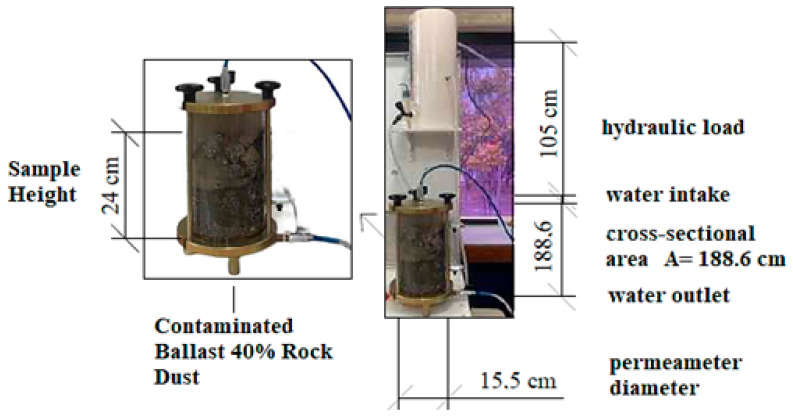
Permeameter with contaminated ballast.

Permeability tests were performed using a constant head permeameter configured as follows: area of 188.60 cm^2^, diameter of 15.50 cm and 105 cm hydraulic load to analyze clean ballast and samples with 10%, 20%, and 40% rock dust fouling and understand the variation in permeability. Figure 1 shows the contaminated ballast inside the permeameter.

A fine mesh screen was placed at the bottom of the device to prevent clogging by fine particles during the test. Next, the volume of material to be tested was placed into the permeameter, supplemented with the previously established percentage of rock dust (0, 10, 20 and 40%), and the lid was closed and sealed. Figure 1 shows the permeameter during the test.

The test results were recorded after circulating water through the sample for 24 h. Two repetitions were carried out for each sample, with five measurements taken at different times considering a 105 cm hydraulic load. Table 2 presents the characteristics of each test.

The test was performed before and after sodium sulfate attack to determine the hydraulic conductivity of the material, simulating the worst possible drainage conditions using particles of the rock itself fragmented by rail operations and local weathering, i.e., saturated conditions.

The hydraulic conductivity (*K*) of the ballast in the present study was calculated using the equation:

(9) $$K=\frac{VL}{Aht}$$

where *V* is the volume of liquid collected; *h* the hydraulic head; *t* test duration; *A* the cross-sectional area of the permeameter; and *L* specimen height.

The Mg1, Mg3, and Gn2 samples were submerged in sodium sulfate anhydrous p.a. (98.5%) purchased locally, representative company of the producing chemical industry. The solution was prepared using 750 g of sodium sulfate (Na_2_SO_4_) per liter of distilled water, according to standard [8], sufficient to achieve saturation at 22 °C.

In accordance with examples cited in the literature [26,27,28], the tests were conducted using predetermined time periods established by [8], which stipulates 18 h of saturation in a sulfate solution and 4 h of oven drying, with 15 min of cooling after drying to prevent thermal shock. Sixty sodium sulfate and drying cycles were used. 

Sodium sulfate attack tests aimed to analyze the effects on rocks used as railway ballast, particularly their permeability, since the high water table level (1.30 m deep) in the coastal region [16] could reach the ballast layer. The heavy rainfall in the area is also an important characteristic to be considered, as reported by [29].

The sodium sulfate attack was conducted in accordance with [8], whereby the samples were immersed in a sodium sulfate solution prepared using 750 g of sodium sulfate anhydrous (Na_2_SO_4_) per liter of distilled water, sufficient to achieve saturation at 22 °C [8].

Testing was carried out using predetermined time periods established by [8], which stipulates 18 h of saturation in a sulfate solution and 4 h of oven drying, with 15 min of cooling after drying to prevent thermal shock. After sample saturation, the material was dried in an oven according to the time period recommended by [8]. Sixty wetting and drying cycles were used.

The chemical attack caused important changes in the rock samples analyzed here. As observed by [30] in sodium sulfate attack on aggregates, there was a gradual increase in hydraulic conductivity and expansion, possibly indicating the development of cracks in the samples, which would explain the increased permeability. This corroborates the importance of investigating the behavior of material under chemical attack.

## 3. Results

### 3.1. Granulometry

**Figure 2:**
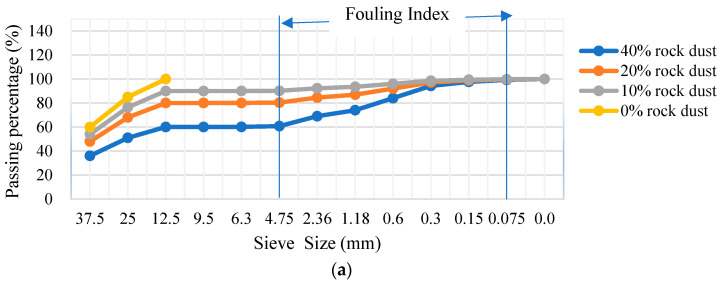

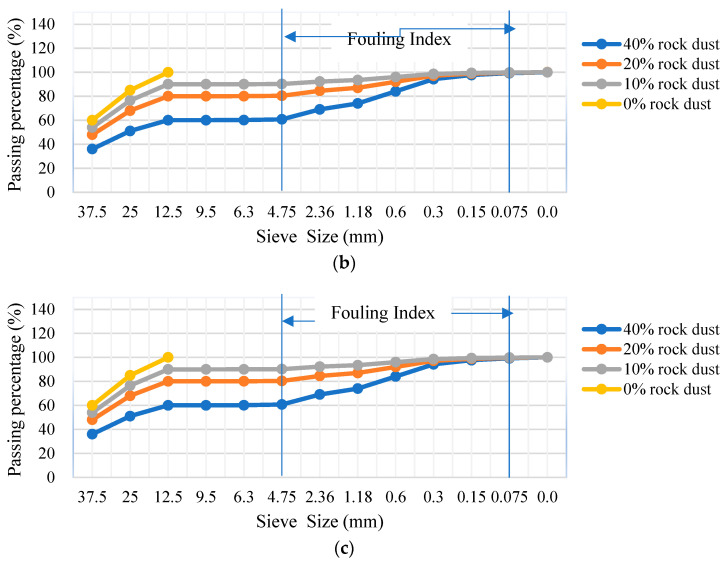
Particle size distribution of clean and fouled ballast with different fouling rates for (**a**) Mg 1, (**b**) Gn 2 and (**c**) Mg 3.

Figure 2 shows the particle size distribution of the clean ballast and ballast with fouling rates of 10%, 20%, and 40% Mg1, Mg3, and Gn2 rock dust, representing the percentage of material that passed through each sieve size. The fouling index (*FI*) [10] defines the level of ballast contamination, where 0 indicates clean ballast, 10% moderately clean, 20% fouled, and 40% highly fouled, indicating the need for immediate cleaning. The different classifications are established according to the value obtained for the index (F_1) (fouling index), which should be calculated based on large mesh sieves. 

Figure 2a–c show the particle size distribution of the metagranites (Mg1 and Mg3) and gneiss with different fouling rates (0%, 10%, 20%, and 40%).

All the material was submitted to sodium sulfate attack, with differences observed in the rock dust after the second decimal place that did not directly affect the particle size of the material degraded by sodium sulfate.

### 3.2. Petrography

**Figure 3:**
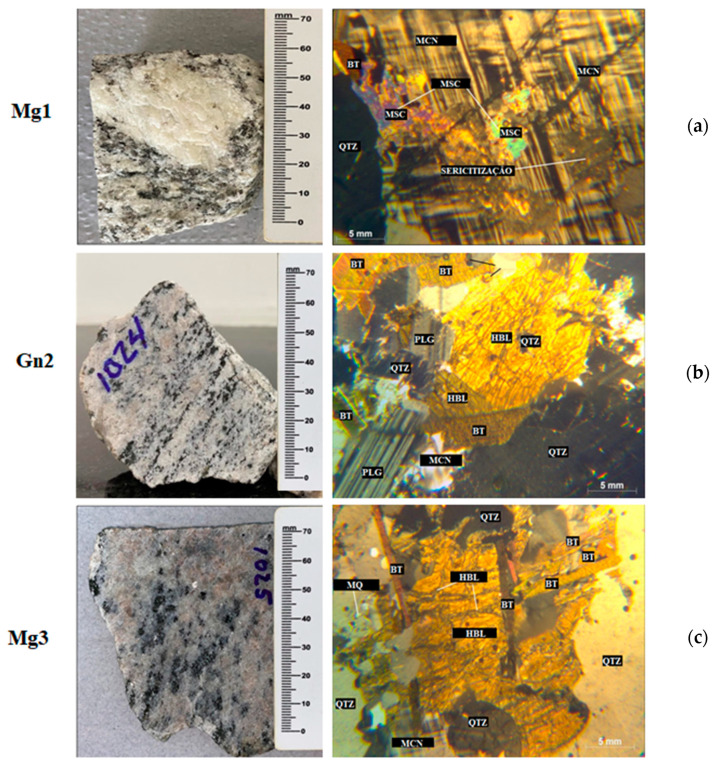
LEFT: Macroscopic view and RIGHT: Microscopic features of the: (**a**) meta granites Mg1; (**b**) gneiss Gn2; (**c**) meta granites Mg3. Crossed Nicols. LEGEND: Qtz—quartz, Plg—plagioclase, Bt—biotite, HBL—hornblende, Mq—mirmequite, Msc—muscovite, Mcn—microcline.

As illustrated in Figure 3, petrographic analyses indicated that all three rocks are metamorphic, with Mg1 and Mg3 exhibiting a non-foliated structure, granoblastic texture, weak to moderate microcracks, little alteration, and irregular points of contact between particles, which are predominantly medium-sized. 

In terms of mineralogical composition, Mg1 is composed of quartz, plagioclase, microcline with protruding and diagonal fissures, biotite, muscovite, and opaque minerals, and Mg3 of quartz, plagioclase, microcline, hornblend, biotite, muscovite, and opaque minerals. The accessory minerals of both the metagranites are zircon and apatite, with defined to undefined grain surfaces. Mg3 also shows a myrmekitic texture with intergrowth of vermicular quartz in plagioclase, preferentially when the latter is in contact with k-feldspar (microcline), as illustrated in Figure 3 (bottom). 

**Figure 4:**
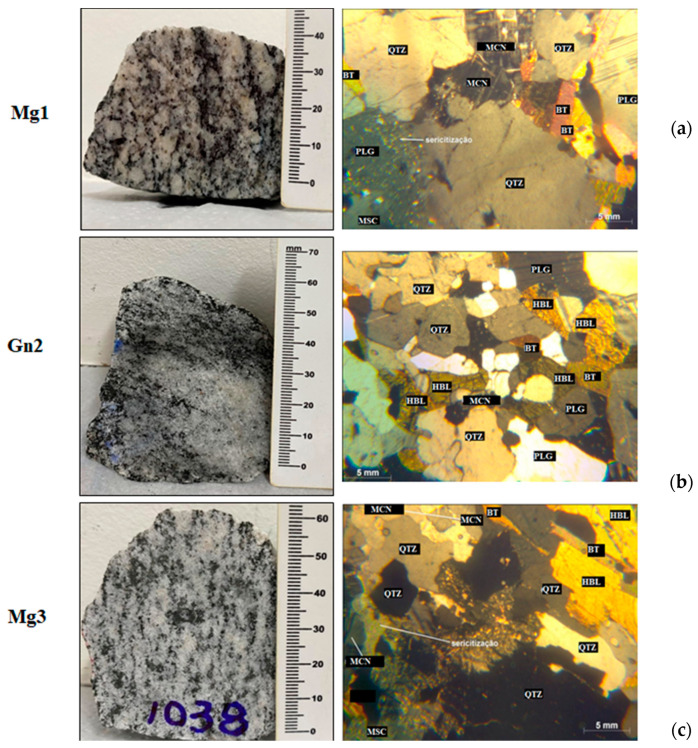
Petrography of the rocks after sodium sulfate attack. LEFT: Macroscopic view and RIGHT: Microscopic features of the: (**a**) meta granites Mg1; (**b**) gneiss Gn2; (**c**) meta granites Mg3. Crossed Nicols. LEGEND: Qtz—quartz, Plg—plagioclase, Bt—biotite, HBL—hornblende, Mq—myrmequite, Msc—muscovite, Mcn—microcline.

The gneiss (Gn2) exhibits an inequigranular grano-lepidoblastic texture, with fine to medium-sized grains varying from hypidiomorphic (some well-defined surfaces) to xenomorphic (no defined surfaces) and irregular contact surfaces between the grains (Figure 4, middle).

Mg1 showed evidence of sericitization in some sections of the rock, with the formation of sericite as a secondary mineral (Figure 3, top). Table 3 presents a summary of the petrographic properties of the rocks.

Mineral alterations after sodium sulfate attack were investigated by macro and microscopic petrographic analyses (Figure 4). A summary of the petrographic properties of the rocks after chemical attack is presented in Table 4, with no significant change in the main component minerals of Mg1, only a 1% zircon reduction. In addition, the minerals present in the material after cycling are not the result of weathering due to sodium sulfate attack, with no change in microcracking, although some quartz crystals were comparatively more microcracked (Figure 3 and Figure 4). Pore size became predominantly medium in relation to the intact rock.

In regard to gneiss, there was a 10% increase in hornblende after sodium sulfate attack, becoming the predominant mineral in the rock, likely due to its natural heterogeneity. Detachment of superficial minerals via accelerated aging and/or the transformation of hornblende into muscovite through weathering were also observed.

In Mg3, a textural change from granoblastic to grano-lepidoblastic was observed, as well as a proportional increase in hornblende due to the heterogeneous and metamorphic nature of the material. Sodium sulfate attack affected both lithologies, since all the samples contain minerals susceptible to weathering such as biotite.

### 3.3. Physical Indices

Testing was performed to assess the physical properties of the rock samples before and after chemical attack. In regard to specific gravity, all the rock samples analyzed complied with [8] prior to chemical attack, with 2.70, 2.66 and 2.67 for Mg1, Gn2 and Mg3, respectively. and were within the 2% porosity limit specified by [8]. Water absorption was 0.60% for Mg1, 0.48% for Gn2 and 0.29% for Mg3.

After 60 sodium sulfate cycles, the samples displayed noteworthy changes, with a greater decline in density and specific gravity for Mg3 due to the presence of minerals susceptible to the 35% sulfate water (15% bt, 15% plg, and 5% msc), as shown in Table 3 based on petrography. This corroborates the increase observed in porosity (from 0.78% to 1.35%) and water absorption (from 0.29% to 0.51%), indicating considerable susceptibility to sulfated water.

Petrographic analysis demonstrated a moderate degree of microcracking in Mg1 after sodium sulfate exposure, with specific gravity of 2.70 g/cm^3^, above that stipulated by [8], porosity of 1.63%, and average pore diameter between 5.47 and 5.80 µm.

Water absorption values for all the samples ranged between 0.51% and 0.69%, in line with those required by [8] of 1.5% and 1%, respectively.

For apparent porosity, Mg1 and Gn2 obtained maximum values above the 2% and 1% stipulated by [8], respectively, with all the rock samples falling within the specified range. When compared to VALEC (2012) guidelines, all the samples obtained porosity greater than 1% and therefore did not meet the requirements. 

After sodium sulfate attack, the porosity and water absorption of all the samples were within the required range. However, Mg3 exhibited higher specific gravity than that recommended by [8] (2.37 g/cm^3^) and considerable susceptibility to weathering.

### 3.4. Rock Porosity–Porosimetry

The different pore sizes of the samples are shown in Figure 3. Gn2 shows greater mercury intrusion into its micropores and varying pore sizes (small, medium, and large), while Mg1 and Mg3 contain no micropores.

In porosity analysis of each intact sample, Gn2 obtained the highest porosity (0.876%) and an average pore diameter of 1.072 µm and Mg3 the lowest (0.338%), with an average pore diameter of 6.097 µm, demonstrating less susceptibility to weathering.

Mg1 and Mg3 displayed less porosity than Gn2 because they have no micropores and undergo less intrusion into small pores. 

**Figure 5:**
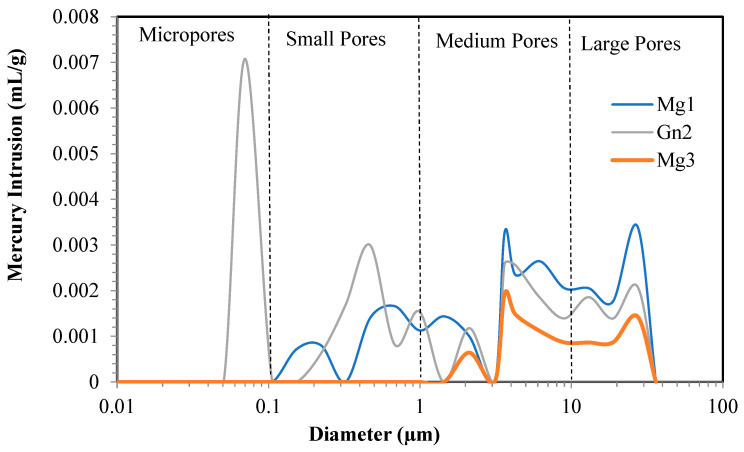
Mercury intrusion porosimetry of Mg1, Mg3 and Gn2.

**Figure 6:**
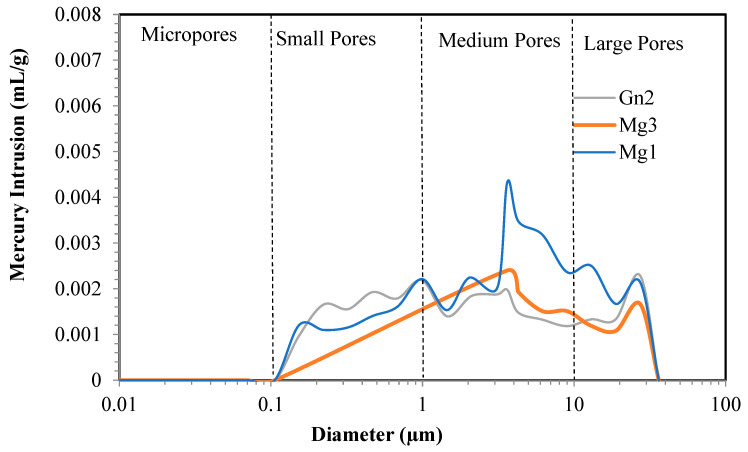
Mercury intrusion porosimetry of Mg1, Mg3 and Gn2 after sodium sulfate attack.

A comparison of Figure 5 and Figure 6 indicates increased intrusion into small and medium-sized pores in Mg1 due to the quartz microcracks observed in petrographic analysis (Figure 4).

Gn2 (Figure 4) showed no micropores after sodium sulfate attack, with smaller microcracks than those in the intact sample (Figure 3). There was no significant difference in average mercury intrusion into small and medium-sized pores.

Mercury intrusion was observed in Mg3 (Figure 6) from small (0.1–1 µm) to large pores (10–100 µm), but Figure 5 shows intrusion into medium to large pores (1–100 µm). This is because chemical attack increased quartz and microcline cracking in the sample (Figure 4) and likely caused leaching and surface detachment in cracked hornblende (Figure 4), with the remaining hornblende less cracked (Figure 4). 

These results corroborate those of [31], who observed an increase in aggregate cracking and crystallization of salt particles within the rock pores, thus increasing stresses in the pores.

A comparison between the results of porosimetry for the samples analyzed and petrographic analyses of the aggregates showed a slight presence of microcracks. Sample Gn2 showed four different pore sizes (micropores, small, medium, and large pores), which can be justified by the presence of microcracks before and after sodium sulfate attack, whereas Mg1 and Mg3 exhibited less porosity despite having medium-sized to large pores. This is because the rock has small pores, no micropores, and fewer large pores, likely due to the presence of clay minerals and iron oxide/hydroxide, which tend to clog the microcracks and reduce porosity. 

### 3.5. Micro–Deval

The Micro-Deval test (MD) indicated greater resistance to mechanical wear in Mg3. Its lower weight loss (8.5 ± 1.27%) may be due to its mineral composition (Table 2) consisting of tougher materials, resulting in less superficial abrasion.

Gn2 showed higher average weight loss (13.28 ± 1.32%), which may be justified by its composition of less tough materials (biotite + muscovite = 44%). This is corroborated by its porosity (0.876%) and pore size (1.072 µm), the highest among the samples analyzed.

**Figure 7:**
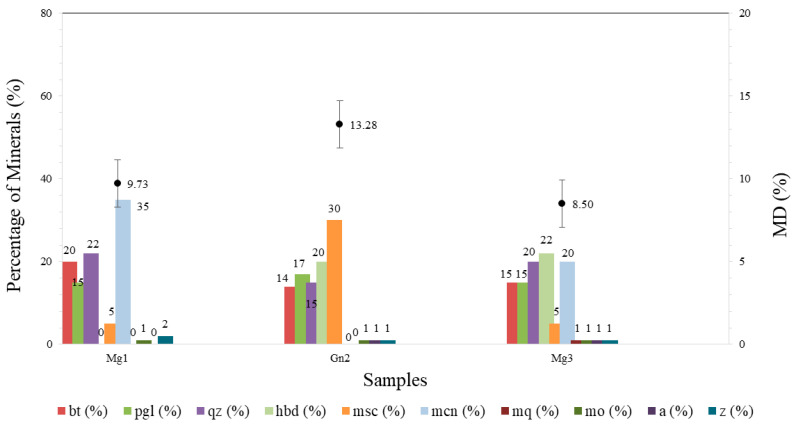
Result of the Micro-Deval test correlated with the mineralogy of the rocks. Hbl: horblende; Qtz: quartz; Plg: plagioclase; Bt: biotite, Msc: muscovite; Mcn: microclimate; Mo: opaque mineral, Z: zircon, A: apatite, C: carbonate.

**Figure 8:**
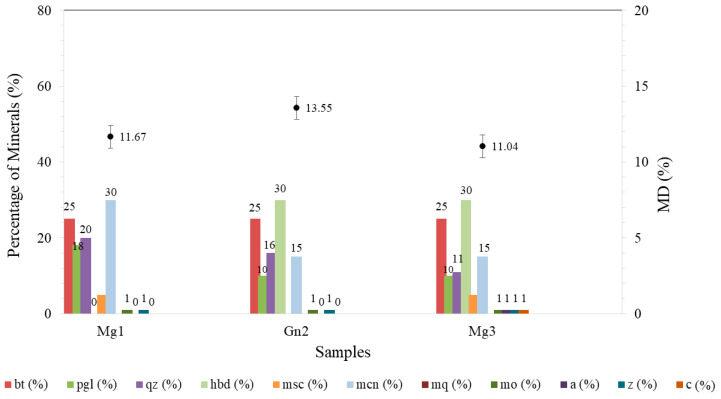
Result of the micro-deval test correlated with rock mineralogy after sodium sufate attack. Hbl: horblende; QTZ: Quartz; PLG: plagioclase; BT: biotite, Msc: muscovite; Mcn: microclimate; Mo: opaque mineral, Z: zircon, A: apatite, C: carbonate.

The average results of the samples in the Micro-Deval test correlated with their mineralogy before sodium sulfate attack, as shown in Figure 7, and after chemical attack in Figure 8.

The Micro-Deval test (MD) was carried out after sodium sulfate attack to assess its effect on the samples and, consequently, the correlation between their component minerals.

Mg3 obtained the largest mean variation in wear (8.5 ± 1.5% to 11.04 ± 0.5%) but continued to show satisfactory resistance to mechanical wear when compared to the other rock types. The greater weight loss is justified by cracks in the hornblende (22%) and the presence of secondary minerals (alteration) in these cracks (Figure 3c), with medium-sized and large pores (Figure 5).

Gn2 (Figure 8) obtained the highest average wear (13.55 ± 0.7%), albeit with no significant difference for this property, since the standard deviations were the same for the intact sample and after chemical attack. In Figure 4b, this sample displayed less cracking after degradation, evident in mercury intrusion, with no significant difference between the intact and degraded samples (Figure 6).

All the samples exhibited a variation in weight loss after the test. Mg1 showed a variation of approximately 1.94% (9.73–11.67%; Figure 8) and Gn2 around 0.27% (13.28–13.55%; Figure 8). The lower mass loss in the latter rock may be due to its mineralogical composition (Table 3), whereby the percentage of hard minerals present led to less surface polishing in the particles. Mg3 displayed the greatest variation among the samples, with 2.54% (8.50–11.04%; Figure 8).

### 3.6. Permeability

Table 5 presents the results of permeability testing for each rock. As rock dust fouling increased, water flow in the permeameter declined, meaning that the hydraulic conductivity of the material decreased.

The hydraulic conductivity rate declined as rock dust fouling increased, as shown in Table 6. Moreover, ref. [6] also reported that the hydraulic conductivity of ballast decreased rapidly as fouling with lateritic soil from the study region increased. For example, the conductivity of 10% fouled ballast was 2.5 × 10^−2^ cm/s and approximately 0.5 × 10^−2^ cm/s for ballast with 40% contamination.

**Figure 9:**
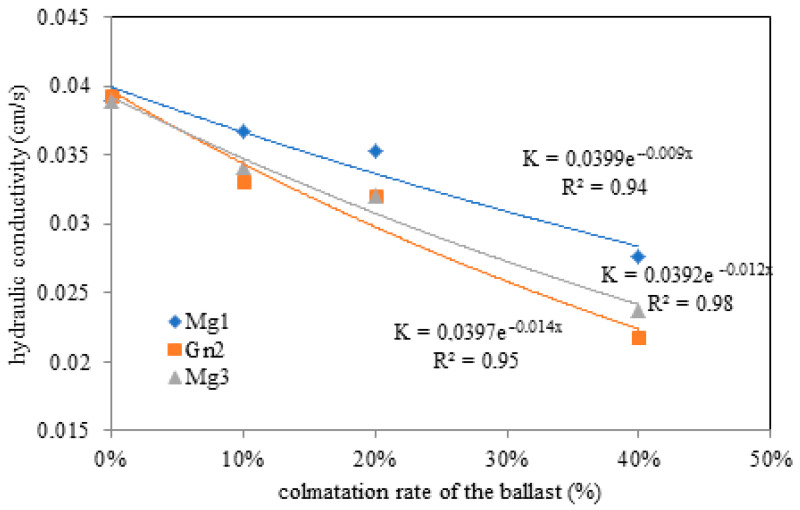
Variation in hydraulic conductivity with ballast fouling rate for Mg1, Mg3 and Gn2.

Hydraulic conductivity values for ballast with 0 (≈4.0 × 10^−2^ cm/s) to 20% fouling were lower than those recorded at 40% fouling. The highest values were observed for Mg1 due to the presence of protruding diagonal cracks in the microcline (Figure 3). The results shown in Figure 9 indicate that hydraulic conductivity varied from fouling onset. 

This finding demonstrates the water drainage issues caused by rock dust fouling in the underlying layers resulting from track loading and local weathering. Figure 9 shows the variation in hydraulic conductivity with fouling in intact ballast. 

The material submitted to sodium sulfate attack also underwent hydraulic conductivity testing to assess its variation, with the results presented in Table 6.

**Figure 10:**
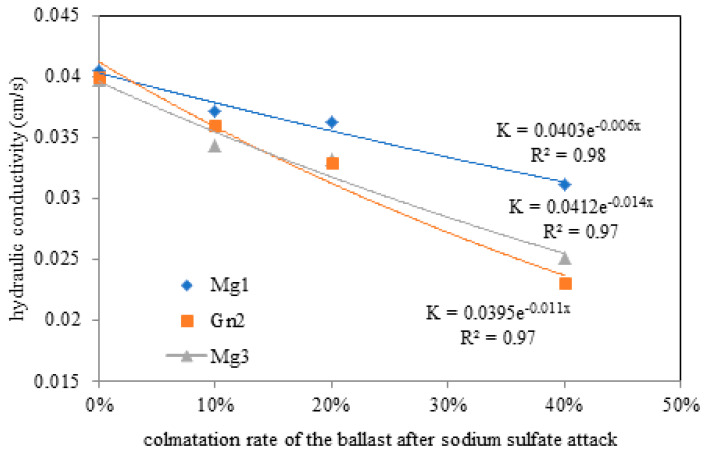
Variation in hydraulic conductivity with ballast fouling rate under sodium sulfate attack in Mg1, Mg3 and Gn2.

The results demonstrate an increase in hydraulic conductivity, even for 0% rock dust fouling, with little change when Figure 9 and Figure 10 are compared. Given that the particle size curves were the same before and after chemical attack (Figure 2), it can be inferred that this difference was influenced by the ballast particles (12.5–50 mm). Differences in hydraulic conductivity were expected based on the results of petrographic analysis (Figure 3 and Figure 4), mercury intrusion porosimetry (Figure 5 and Figure 6), and the variation in wear in MD associated with the mineralogy of each rock assessed (Figure 7 and Figure 8). According to Frazão (2008), sodium sulfate cycling is more harmful to rock because its mechanical strength is linked to its porosity. On the other hand, permeability increased after chemical attack in all the rocks up to 40% rock dust fouling, indicating that, under these specific conditions, the drainage capacity of 10% fouled ballast differs little from that of its clean counterpart, even if the sulfated water reaches the ballast layer. 

The increase in hydraulic conductivity observed here (Figure 10) was also reported by [31] and was directly related to a larger pore diameter, which is the result of salt crystallization within the rock due to chemical attack, exacerbating the stresses generated. 

Figure 10 shows the hydraulic conductivity of the material after sodium sulfate attack.

### 3.7. Sodium Sulfate Attack

The test demonstrated changes in all three samples after sodium sulfate attack, with percentage losses after 60 wetting and drying cycles (in an oven at 110 °C). 

Although there was no difference in texture (Figure 3 and Figure 4), the weight loss observed may be due to the more superficial minerals in the sample, with all values lower than the maximum 10% recommended by [8].

The Mg1 sample showed the greatest weight loss (1.35%) after sodium sulfate attack, while Gn2 and Mg3 obtained 1.00%, confirming that all three samples were susceptible to sulfated water.

## 4. Conclusions

This study analyzed the effect of varying hydraulic conductivity on railway ballast using three types of rock and correlating petrography, physical indices, porosimetry, and hydraulic conductivity before and after sodium sulfate attack based on different rock dust fouling rates to simulate particle wear and abrasion caused by moving trains, which reduces hydraulic conductivity.

The implications of this study in advancing the railway sector in northern Rio de Janeiro state involve characterizing the rocks studied and contributing to decision making in selecting the rock best suited to possible sub-ballast saturation by sulfated water both during construction and track operation.

The results obtained from tests conducted on aggregates extracted from the planned location of the EF-118 railway line help expand the database on these materials and enable the installation of the line in the region based on the range of materials characterized with satisfactory characteristics for railways ballast applications.

Mineralogical analysis of the samples via petrography indicated that all three rock samples (Mg1, Mg3, and Gn2) contain biotite, a mineral susceptible to weathering, which may justify the changes observed after sodium sulfate attack. More microcracks were observed in Mg1, the texture of Mg3 changed from granoblastic to grano-lepidoblastic, and there was an increase in hornblende, a naturally brittle mineral, whereas the mineralogical characteristics of Gn2 remained almost unaltered after chemical attack.

Mercury intrusion porosimetry showed a variation in pore size before and after sodium sulfate attack. In Mg1, quartz microcracking increased intrusion into small and medium-sized pores, whereas Gn2 showed no significant difference in intrusion after attack, with a decline in microcracking in relation to the intact sample. Mg3 exhibited greater pore size variation, varying from medium to large due to sodium sulfate attack. 

Corroborating the results obtained in MD and petrographic analysis, although Mg1 and Mg3 showed greater resistance to mechanical wear, there was also a larger percentage variation in wear after sodium sulfate attack. By contrast, Gn2 obtained the highest among the samples, albeit with no significant variation in average wear. This may be directly related to the mineralogy of the material, since Gn2 remained almost unchanged after sodium sulfate attack.

Permeability testing with different fouling rates showed that hydraulic conductivity declined as ballast fouling increased before and after chemical attack in Mg1, Mg3, and Gn2. There was also a slight increase in hydraulic conductivity after sodium sulfate attack, which maintains ballast integrity in the event of aggregate use. It should be noted that despite the different characteristics and behavior of the samples analyzed under chemical attack, they all obtained satisfactory hydraulic conductivity values. However, it is important to clean ballast with fouling rates of 20% and 40%, classified as fouled and highly fouled. 

In the Gn2 sample, considering the properties analyzed among the tests carried out, the material presented more satisfactory results, verified in addition to favorable physical characteristics, low variation of wear after the abrasion test, and minimal changes after attack by sodium sulfate, which enables its use as railway ballast due to its stability and resistance to wear.

## Figures and Tables

**Table 1:** Particle size distribution of standard ballast—[8].

Sieve Size According to the ABNT	Accumulated Mass Percentage (%)	
mm	Standard A	Standard B
76.2	INAPPLICABLE	0–0
63.5	0–0	0–10
50.8	0–10	INAPPLICABLE
38	30–65	40–75
25.4	85–100	INAPPLICABLE
19	INAPPLICABLE	90–100
12.5	95–100	98–100

**Table 2:** Characteristics of the permeability test.

Test Number	Fouling Rate	Void Ratio	Void Ratio	Void Ratio	Diameter of the Permeameter	Cross-Sectional Area	Length	Sample Height	Water Depth
	(%)	(e)	(e)	(e)	(cm)	(cm^2^)	(h)	(cm)	(cm)
1	0	1.04	1.01	1.02	15.5	188.6	24	24	105
2	0	1.04	1.01	1.02					
3	10	0.93	0.90	0.91					
4	10	0.93	0.90	0.91					
5	20	0.83	0.80	0.81					
6	20	0.83	0.80	0.81					
7	40	0.62	0.60	0.61					
8	40	0.62	0.60	0.61					

**Table 3:** Summary of petrographic analyses of the Mg1/Mg3 meta granites and the Gn2 gneiss. LEGEND: Hbl-horblende; Qtz-quartz; Plg-plagioclase; Bt-biotite, Msc-muscovite; Mcn: microcline; Mq: myrmequite, Mo: opaque mineral.

Type	Color	Grain Size	Texture	Microcracking	Alteration	hbl	Qtz	Plg	Bt	Msc	Mcn	Mq	Mo
Mg1	Grayish White	Fine (5%), Medium (50%) Coarse (45%)	Granoblastic	Moderate	Little	-	22	15	20	5	35	-	-
Gn2	Whitish Gray	Fine (20%) Medium (80%)	Granolepidoblastic	Weak	Little	20	15	17	14	30	15	-	-
Mg3	Pink Gray	Medium (80%) Fine (20%)	Granoblastic	Weak	Little	22	20	15	15	5	20	1	1

**Table 4:** Summary of petrographic analysis results for meta granites Mg1/Mg3 and gneiss Gn2 after sodium sulfate attack. LEGEND: Hbl-horblende; Qtz-quartz; Plg-plagioclase; Bt-biotite, Msc-muscovite; Mcn-microcline; Mq—myrmequite, Mo—opaque mineral.

Typeo	Color	Grain size	Texture	Microcracking	Alteration	hbl	Qtz	Plg	Bt	Msc	Mcn	Mq	Mo
Mg1	Gray	Fine (10%), Medium (70%) Coarse (20%)	Grano-lepidoblastic	Moderate	Little	-	20	18	25	5	30	-	1
Gn2	Grayish White	Fine (30%) Mediium (70%)	Grano-lepidoblastic	Weak	Little	30	16	10	25	2	15	-	1
Mg3	Gray	Medium (80%) Fine (20%)	Grano-lepidoblastic	Weak	Little	30	11	10	25	5	15	-	1

**Table 5:** Average hydraulic conductivity values for each colmatation rate (0 to 40%) of the ballast of Rocks Mg1, Mg3 and Gn2 of intact rocks.

Rock	Ballast Fouling Rate (%)	Hydraulic Conductivity (cm/s)
Mg1	0	0.03
	10	0.03
	20	0.03
	40	0.02
Gn2	0	0.03
	10	0.03
	20	0.03
	40	0.02
Mg3	0	0.03
	10	0.03
	20	0.03
	40	0.02

**Table 6:** Hydraulic conductivity values for each ballast fouling rate after sodium sulfate attack in Mg1, Mg3 and Gn2 rock samples.

Rock	Ballast Fouling Rate (%)	Hydraulic Conductivity (cm/s)
Mg1	0	0.04
	10	0.37
	20	0.03
	40	0.03
Gn2	0	0.03
	10	0.03
	20	0.03
	40	0.02
Mg3	0	0.03
	10	0.03
	20	0.03
	40	0.02

## Data Availability

Not applicable.
